# Critical Evaluation of the Lund Concept for Treatment of Severe Traumatic Head Injury, 25 Years after Its Introduction

**DOI:** 10.3389/fneur.2017.00315

**Published:** 2017-07-04

**Authors:** Per-Olof Grände

**Affiliations:** ^1^Anesthesia and Intensive Care, Department of Clinical Sciences Lund, Faculty of Medicine, Lund University, Lund, Sweden

**Keywords:** brain injury, intracranial monitoring, neuroinflammation, neuroradiology, neuro-intensive care, the Lund concept, the penumbra zone, brain perfusion

## Abstract

When introduced in 1992, the Lund concept (LC) was the first complete guideline for treatment of severe traumatic brain injury (s-TBI). It was a theoretical approach, based mainly on general physiological principles—i.e., of brain volume control and optimization of brain perfusion and oxygenation of the penumbra zone. The concept gave relatively strict outlines for cerebral perfusion pressure, fluid therapy, ventilation, sedation, nutrition, the use of vasopressors, and osmotherapy. The LC strives for treatment of the pathophysiological mechanisms behind symptoms rather than just treating the symptoms. The treatment is standardized, with less need for individualization. Alternative guidelines published a few years later (e.g., the Brain Trauma Foundation guidelines and European guidelines) were mainly based on meta-analytic approaches from clinical outcome studies and to some extent from systematic reviews. When introduced, they differed extensively from the LC. We still lack any large randomized outcome study comparing the whole concept of BTF guidelines with other guidelines including the LC. From that point of view, there is limited clinical evidence favoring any of the s-TBI guidelines used today. In principle, the LC has not been changed since its introduction. Some components of the alternative guidelines have approached those in the LC. In this review, I discuss some important principles of brain hemodynamics that have been lodestars during formulation of the LC. Aspects of ventilation, nutrition, and temperature control are also discussed. I critically evaluate the most important components of the LC 25 years after its introduction, based on hemodynamic principles and on the results of own an others experimental and human studies that have been published since then.

## Introduction

The principles of the Lund concept (LC) for treatment of severe traumatic brain injury (s-TBI) (Glascow Coma Score 3–8) were introduced in 1992 at the Swedish Society of Anesthesia congress in Lund (1), with a first clinical study published in 1994 (2). It was a new, controversial approach, mainly based on physiological and pathophysiological principles of brain volume regulation and perfusion of the most injured parts of the brain. The experience from 25 years of use supports the view that the LC appears to be beneficial not only for the brain but also for other organs of the body, such as the lung. Treatment with the LC is primarily based on the pathophysiological mechanisms behind symptoms rather than just treating the symptoms. A comprehensive presentation of the principles and guidelines of the LC has already been published (3). It is reasonable to assume that making the hypoxic penumbra zone of the injured brain to survive by optimizing brain perfusion and oxygenation is crucial when discussing outcome of the brain. Thus, apart from counteracting an increase in intracranial pressure (ICP), the LC is also aimed at improving microcirculation and oxygenation of the hypoxic penumbra zone and can, therefore, be classified as an *ICP and perfusion-targeted therapy*.

In principle, the current concept does not differ much from the one originally presented, except that the venoconstrictor dihydroergotamine has been phased out. Dihydroergotamine was initially used in patients with a refractory, life-threatening high ICP, but its use was discontinued because of potential adverse peripheral vasoconstrictor effects.

The LC resulted in treatments that appeared to be quite contrary to the alternative ideas presented a few years later. Alternative guidelines for treatment of s-TBI, e.g., the Brain Trauma Foundation guidelines and European guidelines (4, 5), were mainly based on meta-analytic approaches and systematic reviews, originating from clinical outcome studies. The LC was severely criticized by advocates of the alternative guidelines, but in the intervening 25 years the LC has gained more acceptance. Continuous ICP recording is recommended in both the LC and the alternative s-TBI guidelines.

The LC differs from other guidelines not only regarding therapeutic components but also regarding the time to start the therapy. In the alternative guidelines, the ICP-reducing therapy should start when ICP is above 20 mmHg (4, 6), a value that has been increased to 22 mmHg in the latest update from the Brain Trauma Foundation (7). In contrast, the LC recommends that the therapy should start early after arrival at the hospital irrespective of prevailing ICP (3, 8). This will counteract an increase in ICP prophylactically from the start.

Even though the LC on the one hand and the alternative guidelines on the other still differ from each other in essential respects, the Brain Trauma Foundation guidelines have moved closer to the LC in the past 10–15 years, e.g., concerning cerebral perfusion pressure (CPP) and more strict use of vasopressors and mannitol (6) (Table 1).

**Table 1:** Examples of components where the BTF guideline has approached or deviated from the Lund concept (LC).

LC	BTF at its introduction (1996)	BTF 2007/2016
Cerebral perfusion pressure (CPP) 50–70 mmHg	CPP above 70 mmHg	CPP 50–70 mmHg
Avoidance of osmotherapy	Osmotherapy a main intracranial pressure-reducing therapy	Osmotherapy still used, but with more caution
Avoidance of vasopressors	High doses of vasopressors accepted to keep CPP above 70 mmHg	Vasopressors can be used, but less frequent to avoid ARDS
Active cooling is not used	Active cooling is accepted	Active cooling is not used
Albumin recommended as plasma volume expander	Albumin recommended as plasma volume expander	Albumin not specifically recommended

Several smaller, single-center outcome studies using the LC have shown good outcome (9–11). Two smaller randomized studies compared a modified version of the LC with a more conventional CPP-targeted treatment (12, 13). Both of these studies showed significantly better outcome with the modified LC than with the conventional guideline. All outcome studies using the LC have been summarized and discussed in a recent review (8).

A study by Patel et al. (14) evaluated trends in outcome in head-injured patients who underwent conventional treatments in England and Wales between 1989 and 2003. They found no improvement in outcome during that period. This conclusion has been supported by two recent reviews (15, 16), which showed that modern research has not improved outcome following TBI, although progress has been made in understanding the pathophysiology of s-TBI and the general hospital care has been improved. A study of s-TBI patients, in which the patients were treated according to the Brain Trauma Foundation guidelines, did, however, show improvement in outcome between 2001 and 2009 (17).

Apparently, we still lack convincing evidence-based support for many of the treatments used in various s-TBI guidelines, and this in spite of the fact that several randomized studies have been performed evaluating specific components (14, 15), such as effect of hypothermia, or any of the pharmacological treatments tested. Further, there is no large randomized study performed comparing the overall outcome from different s-TBI guidelines. From this point of view, all guidelines are equally deficient. A specific therapy, therefore, to a large extent must be based on other types of input such as smaller clinical outcome studies, experimental studies, basal physiological principles, systematic reviews, and meta-analytic approaches.

To understand and evaluate the different components of the LC, one must be familiar with its theoretical background. In the present review, I describe some basic physiological and pathophysiological mechanisms controlling brain volume and brain perfusion, which have been lodstars during formulation of the LC. “Nature knows best” is a motto of the LC, and it strives for normality for most hemodynamic and ventilatory parameters—as well as normality regarding electrolytes, temperature, nutrition, and stress. Experimental and human studies from our group and other groups evaluating the different components of the LC will be presented in this review, and used for a critical evaluation of the therapeutic components of the LC. I also speculate about possible not proven physiological explanations of some well-known, but still not fully understood, scenarios in s-TBI patients.

## Principles for Brain Volume Regulation

As the brain is enclosed in a rigid cranium with only minor space for intracranial expansion, the brain volume must be kept at a relatively constant level to avoid adverse alterations in ICP. The normal brain, therefore, has a more sophisticated volume regulation than that of other organs of the body.

**Figure 1:**
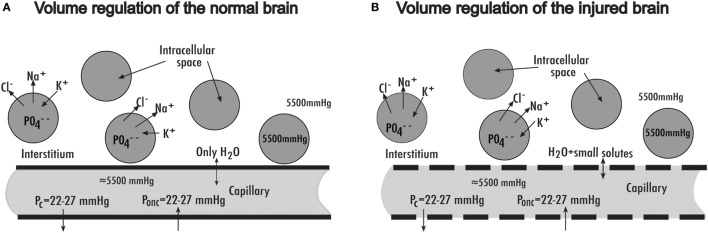
**(A)** Schematic illustration of a cerebral capillary in the normal brain with intact blood–brain barrier (BBB), also showing the Starling forces (transcapillary hydrostatic and oncotic pressures) responsible for transcapillary fluid exchange. Only water can pass through the intact BBB passively. **(B)** Cerebral capillary and the Starling forces in the injured brain with disrupted BBB, in which the capillaries are passively permeable to water and small solutes. For more details of the volume regulation mechanisms in the normal and injured brain, see text. Reproduced from Ref. (3), with permission.

In all organs outside the brain, the capillaries are passively permeable to smaller molecules such as Na^+^ and Cl^−^ ions, and to some extent also to larger macromolecules, such as proteins. The capillaries of the normal brain differ from those of the rest of the body, in the sense that they are passively permeable to water only, a feature that characterizes the intact blood–brain barrier (BBB) (Figure 1A) (18). Electrolytes and larger molecules cannot cross the intact BBB in a passive way. In the injured brain, on the other hand, especially in the most injured parts of the brain, the BBB is disrupted, which means that electrolytes (but not larger molecules) can pass passively (Figure 1B). Thus, the passive permeability to proteins and other macromolecules of cerebral capillaries in both the normal and the injured brain is very low, as indicated by the small increase in protein concentration in cerebrospinal fluid (CSF) of only 0.5–2 g/L from values of 0.5–1 g/L after an s-TBI, as compared to a normal protein concentration in plasma of 60–70 g/L.

An imbalance of the Starling fluid equilibrium in the brain—in terms of an increase in transcapillary hydrostatic capillary pressure, e.g., after an increase in arterial pressure, or a decrease in transcapillary oncotic pressure—will start filtration (Figure 1A). If the BBB is intact, the filtrate consists only of water. This means that the filtration will cease very soon, as water filtered to the interstitium decreases the interstitial crystalloid osmotic pressure by dilution from its normal value of about 5,500 mmHg, creating an absorbing crystalloid osmotic counter-pressure. This explains why an intact BBB is essential to maintain brain volume at a relatively constant level (18).

A disrupted BBB, on the other hand, means that following an imbalance in the Starling fluid equilibrium toward filtration, the filtration will continue, as the filtrate has about the same crystalloid composition as the interstitium (Figure 1B). Thus, the filtrate will cause no or just a small amount of interstitial osmotic dilution, and no or just a small amount of absorbing osmotic counter-pressure will develop. The filtration will, therefore, continue, creating a vasogenic brain edema, until it is successively counteracted and stopped by the subsequent increase in ICP. According to these principles, the vasogenic brain edema can develop only if there is an imbalance in the Starling fluid equilibrium and a simultaneous passive permeability to small solutes (e.g., Na^+^, Cl^−^).

Because of impaired autoregulatory capacity in the injured brain, partly due to impaired myogenic capacity, there is an increase in hydrostatic capillary pressure, which will be gained by an increase in the arterial inflow pressure. Realistic values of the imbalance in the Starling equation created by the increase in transcapillary hydrostatic pressure when autoregulation is impaired can be estimated to be 4–5 mmHg at most.

At first sight, it seems paradoxical that the vasogenic brain edema can result in a much higher increase in ICP than the initial increase in transcapillary hydrostatic capillary pressure. This “*paradoxical event*,” however, can be explained from hemodynamic principles for an organ enclosed in a rigid shell and will be discussed in more detail below under Section “Hemodynamic Consequences of the Rigid Cranium.”

Most likely, various permeability-increasing proinflammatory substances released after a head trauma are involved in the disruption of the BBB. While a disrupted BBB is essential for the development of a vasogenic extracellular brain edema, the cytotoxic brain edema is mainly intracellular—caused by damage to cell membranes, e.g., from hypoxia and various toxic substances, such as cytokines and free radicals (19, 20). Magnetic resonance imaging has shown that intracellular edema is part of posttraumatic brain swelling, and occurs mainly around contusions (21). Mitocondrial dysfunction is also suggested as an important pathophysiological mechanism after an s-TBI. Several proinflammatory substances released after brain trauma have been evaluated as substances responsible for development of cell injury and brain edema (22). All studies so far that have tested antagonists of some of the toxic substances discussed, have, however, failed to improve outcome (22–24).

Irrespective of types of substances released, hypoxia is presumed to be an important triggering mechanism and cause of secondary injury to the brain (25, 26). As the most injured parts of the brain suffer from severe hypoxia, improvement of perfusion and oxygenation of these areas should help to reduce the cytotoxic brain edema, a hypothesis adopted in the LC. Hypoxia may also increase brain edema by increased interstitial osmotic pressure from cellular and molecular disintegration inducing transcapillary filtration (27). Consequently, the main parts of both the vasogenic and the cytotoxic brain edema will develop in the most injured parts of the brain.

## Hemodynamic Consequences of the Rigid Cranium

**Figure 2:**
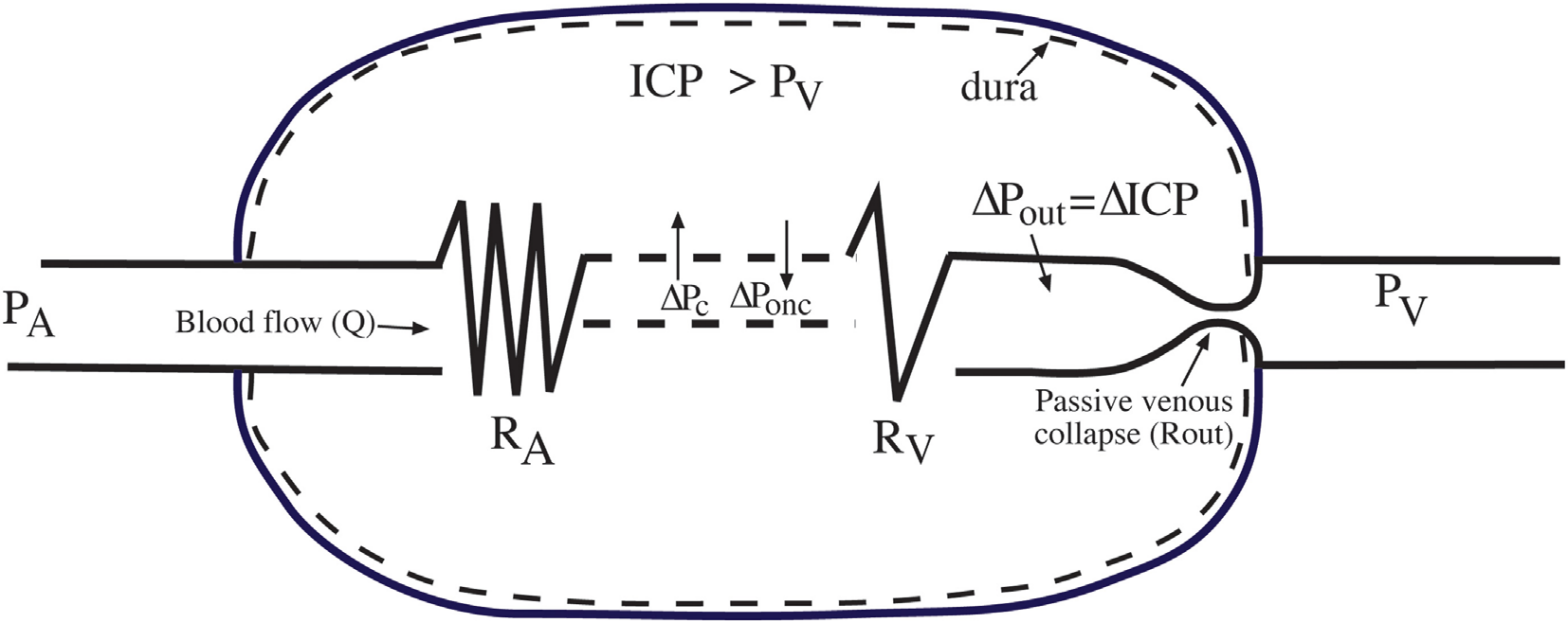
Hemodynamic consequences for the brain enclosed in the rigid dura/cranium. Δ*P*c, transcapillary hydrostatic capillary pressure; Δ*P*_onc_, transcapillary oncotic pressure; *P*_A_, the arterial inflow pressure; Q, cerebral blood flow; *R*_A_, arterial precapillary resistance; *R*_V_, venular resistance; Δ*P*_out_, transvascular pressure retrogradely to the subdural venous collapse (*R*_out_); *P*_V_, is extracranial venous pressure. For details, see text. Reproduced from Ref. (3), with permission.

Important hemodynamic characteristics of the brain result from the fact that it is enclosed in a rigid cranium. Some of these characteristics will be discussed below from the schematic illustration of the brain circulation enclosed in a rigid shell shown in Figure 2.

Intracranial pressure for the normal brain is 8–11 mmHg, and it is kept at this level by a balance between production and consumption of CSF. This can be compared with normal tissue pressure in the rest of the body of 0 to −2 mmHg (28). The brain is the only organ of the body with a significantly positive tissue pressure. This is not a coincidence, as a positive ICP is essential for proper function of the normal brain, as explained below.

The venous pressure outside the dura (*P*_V_ in Figure 2) is close to 0 or even negative at upright position. This means that there is a pressure fall in the veins between the subdural and the extradural space. This pressure fall has—somewhat inappropriately—been called a waterfall phenomenon (29–32). As early as 1928, it was shown experimentally (33), that this pressure fall creates a venous collapse at a short distance before the veins leave the brain, creating a subdural venous outflow vascular resistance (*R*_out_ in Figure 2). As venous pressure just before *R*_out_ (*P*_out_ in Figure 2) will change in parallel with the variation in ICP, the resistance created by the passive collapse (*R*_out_) is directly related to the pressure fall ICP-*P*_V_ (Figure 2) (31, 34).

The existence and hemodynamic consequences of a variable collapse for an organ enclosed in a rigid shell was confirmed and analyzed experimentally on a normally perfused cat skeletal muscle enclosed in a closed plethysmograph (30, 31). It is relevant to simulate hemodynamics of the brain related to the rigid cranium on a skeletal muscle enclosed in a plethysmograph, as the results are general for any organ enclosed in a rigid and closed shell (30–32).

**Figure 3:**
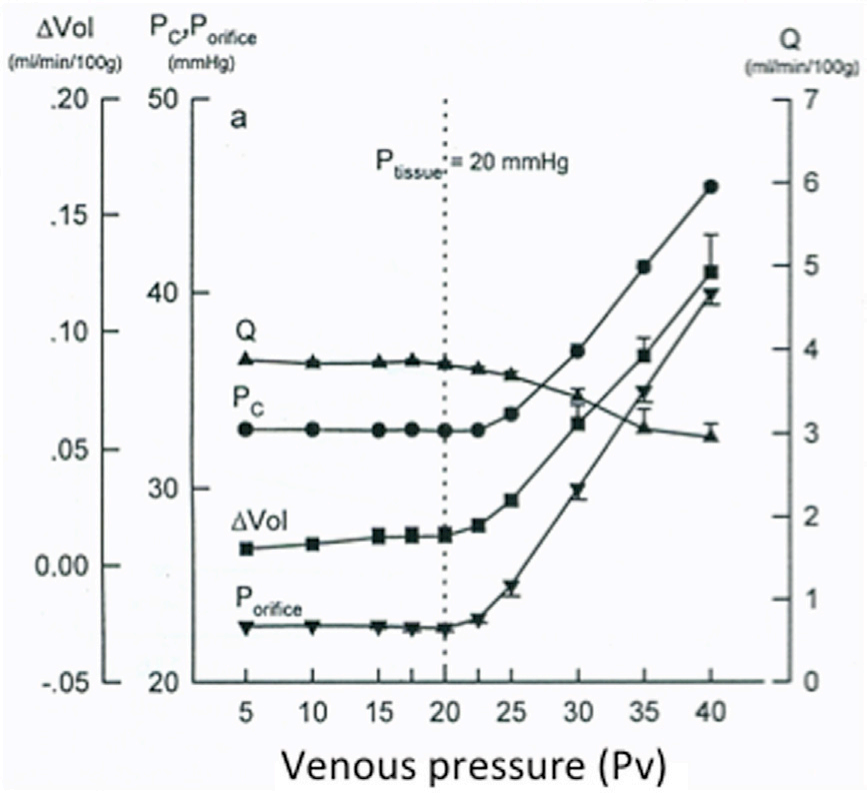
Hemodynamic effects of an organ (skeletal muscle) enclosed in a rigid shell (plethysmograph) with a tissue pressure of 20 mmHg of variation in venous pressure just outside the shell (*P*_V_). The experiment was intended to simulate the attention: hemodynamic effects of extradural *P*_V_ variations in the brain enclosed in the dura/cranium. The figure shows effects of change in *P*_V_ on hydrostatic capillary pressure (*P*c), on venous pressure just upstream of the venous collapse (*P*_orifice_ = *P*_out_ in Figure 2), on change in tissue volume (ΔVol), and on blood flow (Q). Note that there are no hemodynamic effects of change in *P*_V_ if *P*_V_ is below the tissue pressure. If *P*_V_ is above tissue pressure, there is no protective venous collapse (no *R*_out_, Figure 2), and the variations in *P*_V_ are transferred in retrograde manner to the skeletal muscle. Reproduced from Ref. (30), with permission.

Figure 3 shows some hemodynamic parameters from such an experimental model. As seen, *P*_orifice_ (corresponding to *P*_out_ in Figure 2), tissue volume (Vol), and blood flow to the organ (Q) do not change when venous pressure (*P*_V_ in Figure 3, corresponding to *P*_V_ in Figure 2) varies, as long as *P*_V_ is below the tissue pressure (*P*_tissue_) of 20 mmHg. An increase in *P*_V_ when *P*_V_ is above *P*_tissue_ (there is no protecting venous collapse) results in an increase in *P*_orifice_ (*P*_out_ in Figure 2), an increase in hydrostatic capillary pressure (*P*_c_) and increase in tissue volume (Vol), while blood flow (Q) is reduced due to reduction in perfusion pressure. That *P*_c_ increases in parallel with an increase in *P*_tissue_ is supported by Figure 3—showing a *P*_c_ of about 34 mmHg at a *P*_tissue_ of 20 mmHg compared to a normal *P*_c_ in a skeletal muscle of 15 mmHg. It can be concluded from Figure 3 that the variable passive venous collapse will protect the brain from extracranial venous pressure (*P*_V_) variations as long as *P*_V_ is lower than ICP.

The existence of a passive sudural venous collapse and its hemodynamic consequences also finds indirect but strong support, for two reasons. First, what would happen in our daily lives if a subdural venous resistance protecting the brain from venous pressure variations did not exist? If this was the case, there would be a drastic reduction in intracranial venous blood volume (70–80% of the intracranial blood volume is situated on the venous side of the brain), with marked hemodynamic consequences when changing from supine position to upright position and *vice versa*. An extracranial venous collapse when changing from supine to upright position might also help to protect the brain from variations in venous pressure.

Second, calculation of CPP as arterial pressure (*P*_A_) minus ICP and not minus *P*_V_, as done when calculating the perfusion pressure in other organs of the body, can be entirely explained by the existence of a variable passive subdural outflow resistance (*R*_out_). Thus, CPP is *P*_A_ minus the pressure just upstream of *R*_out_, which is the same as ICP (Figure 2). This means that by accepting CPP as *P*_A_ − ICP, we also have to accept a passive variable subdural venous resistance compensating for extradural variations in *P*_V_ (Figures 2 and 3). Note that if *P*_V_ is higher than ICP, there is no protecting venous collapse and perfusion pressure should be calculated as *P*_A_ − *P*_V_.

As mentioned under Section “Principles for Brain Volume Regulation” above, the increase in ICP from a vasogenic brain edema may be much greater than the disturbance in the Starling fluid equilibrium following an increase in hydrostatic capillary pressure and decrease in plasma oncotic pressure. This paradoxical event has been described previously (34) and will be commented on below.

Imbalance between hydrostatic and oncotic transcapillary pressures in the injured brain with a disrupted BBB will start filtration and slowly increase ICP. The simultaneous increase in *R*_out_ due to the increase in ICP − *P*_V_ means a similar increase in *P*_out_, which will be transferred in a retrograde manner to the capillaries (Figure 2). This will result in an increase in *P*_c_, which will cause further filtration and further increase in ICP, and so on. A new steady state at a raised ICP will finally be established. Due to a fall in pressure in venules of about 20%, only about 80% of the increase in ICP is transferred in retrograde manner to the capillaries. This means that the highest increase in ICP by a vasogenic brain edema at steady state will be eight times larger than the initial imbalance between transcapillary hydrostatic pressure (*P*_c_) and the transcapillary oncotic pressure (*P*_onc_) (34). This mechanism explains why the increase in ICP caused by vasogenic brain edema can be much larger than the initial increase in *P*_c_ and decrease in *P*_onc_.

Consequently, a reduction in *P*_c_—for example, by antihypertensive therapy—may reduce ICP caused by a vasogenic edema at most by eight times more than the initial decrease in *P*_c_. This physiological scenario strengthens the motivation for using antihypertensive therapy in head-injured patients with raised ICP (see under “Blood pressure” below).

**Figure 4:**
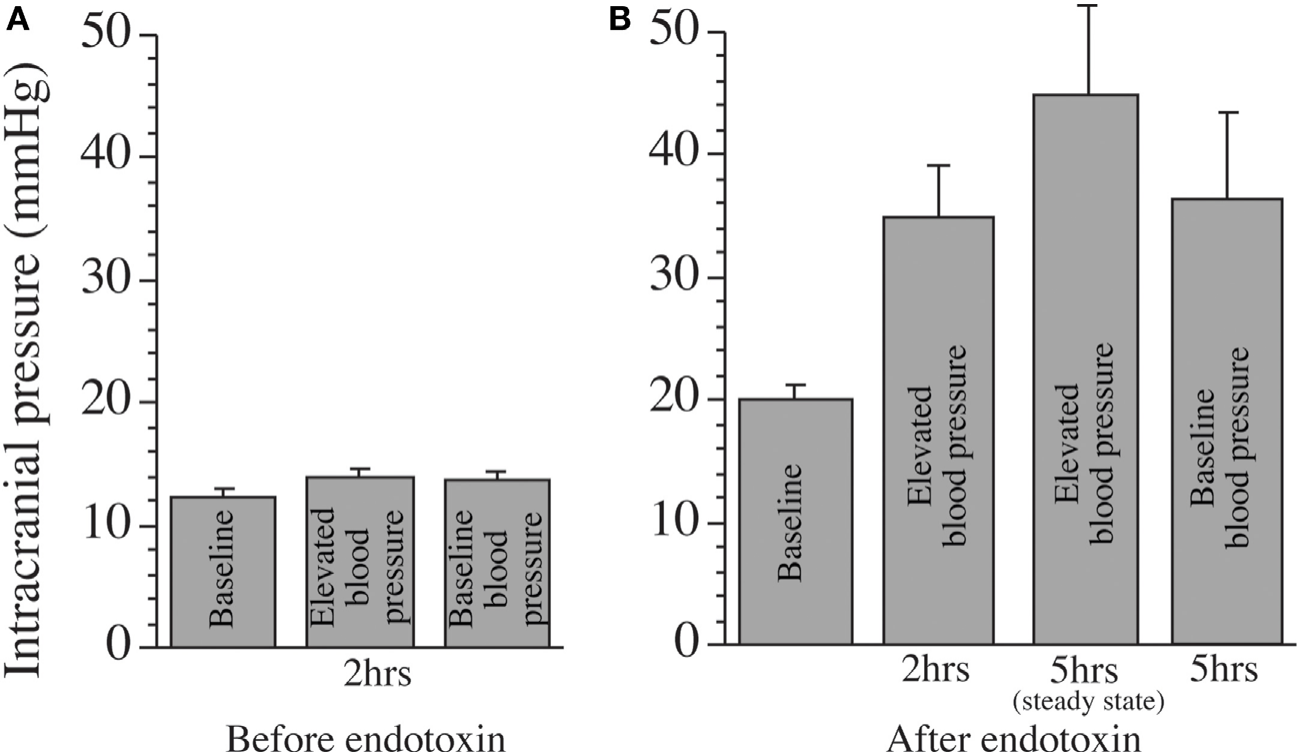
Effects of an increase in mean arterial blood pressure of 30 mmHg, by intravenous infusion of dobutamin and angiotensin II, on intracranial pressure (ICP) in the cat. **(A)** ICP with intact blood–brain barrier (BBB) with normal arterial blood pressure (baseline) and after 2 h of elevated blood pressure (by about 30 mmHg). ICP was not affected by the increase in blood pressure after 2 h and after 5 h (5-h data are not shown). There was no change in ICP when mean arterial pressure returned to baseline. **(B)** ICP after disruption of the BBB with intrathecal endotoxin infusion at normal arterial blood pressure (baseline) and after 2 and 5 h with increased mean arterial blood pressure by about 30 mmHg. There was a significant increase in ICP by about 25 mmHg from the increase in arterial pressure. ICP was slightly reduced shortly after the arterial blood pressure returned to baseline, an effect most likely due to a decrease in intracranial blood volume. As can be seen, this effect was smaller from a normal ICP **(A)** than from a higher ICP **(B)**, due to the unlinear pressure–volume curve for the brain. Reproduced from Ref. (35), with permission.

The hypothesis of a much greater increase in ICP than the initial imbalance in the Starling equilibrium has found support in a study on the cat (35), the results of which are presented in Figure 4. It showed, as expected, that ICP was not affected by an increase in mean arterial pressure of 30 mmHg by infusion of angiotensin II and dobutamin when the BBB was intact (Figure 4A). When the BBB was disrupted, accomplished by intrathecal endotoxin infusion, there was an increase in ICP of 25 mmHg at steady state 5 h after a similar increase in arterial pressure of 30 mmHg (Figure 4B). The marked increase in ICP in this study supports the importance of the blood pressure for development of a vasogenic brain edema at a disrupted BBB and also the hypothesis that ICP can increase much more than the initial increase in *P*_c_.

Some clinical consequences for the brain of the existence of a variable passive subdural venous collapse will be presented below. For example, the decrease in extracranial venous pressure by head elevation causes a corresponding increase in *R*_out_, preventing the extradural venous pressure decrease from being transferred in retrograde manner to the brain (31). There will, therefore, be no increase in venous drainage from the brain (i.e., no decrease in intracranial venous blood volume) after head elevation, as has also been suggested previously (36, 37) (cf. Figure 3). The immediate decrease in ICP after head elevation can instead be explained by passive decrease in intracranial blood volume on the arterial side, when arterial pressure to the brain is reduced.

Most probably due to the suggested risk of increase in venous pressure and ICP by positive end-expiratory pressure (PEEP), PEEP is not recommended as an obligatory therapy in alternative guidelines (5–7). The physiological theories presented above, however, contradict the idea of such a risk—as the increase in extracranial venous pressure by PEEP will not be transferred to the brain. This hypothesis found support in a clinical study by Caricato et al., which showed that PEEP increased central venous pressure and jugular pressure without affecting ICP (38). Thus, PEEP (normally 6–8 cmH_2_O) has been mandatory in the LC to protect the lung from atelectasis and ARDS. PEEP should always be below ICP to be sure that the subdural outflow resistance (*R*_out_ in Figure 2) is preserved. By now results of several studies have supported the use of PEEP in TBI patients (38–41).

The normal brain is protected from variations in arterial pressure through a balance between the active myogenic and the metabolic control systems, a phenomenon called autoregulation. The myogenic control system is vulnerable, which explains why the true autoregulation of blood flow is significantly depressed after a brain trauma. The degree of autoregulation for the whole brain is also affected negatively by the fact that blood flow in the contusion areas is very low and that these areas lack autoregulation. Impaired autoregulation in less injured parts of the brain, mainly through impared myogenic reactivity, is probably of less importance for outcome—as impaired myogenic reactivity means vasodilation.

In summary, the normal brain is protected from arterial pressure variations *via* a myogenically active mechanism called autoregulation of blood flow, which is depressed after a head injury. Both the normal and the injured brain are protected from venous pressure variations *via* a passive mechanism based on the existence of a variable venous outflow resistance.

## Blood Pressure and CPP

Hypertension is common after a head injury, most likely due to a hyperadrenergic state (42–45). If arterial blood pressure is low after an s-TBI without external bleeding, in most cases it is an effect of hypovolemia. The purpose of maintaining a high CPP in the alternative guidelines was to prevent cerebral ischemia by squeezing oxygenated blood through the swollen brain (CPP-targeted therapy) (4, 5, 46). This has been a generally accepted concept in most of the alternative guidelines. A human study by Simard and Bellefleur (47) and a rabbit study by Durward et al. (48) raised questions regarding this concept.

A supranormal CPP was criticized in the LC from the start, based on physiological aspects of brain volume regulation of the injured brain as described above and also by adverse effects of vasopressors. Thus, a main component of the LC is the use of antihypertensive therapy with the purpose of reducing arterial pressure, cerebral hydrostatic capillary pressure, and adrenergic stress, which are all elevated after a head injury. Only antihypertensive drugs that do not induce a simultaneous cerebral vasodilation are used, as cerebral vasodilation will increase hydrostatic capillary pressure, intracranial blood volume, and ICP. If a marked brain edema and high ICP have already developed, antihypertensive therapy will reduce brain edema, but this is a slow process, most likely due to a relatively low water permeability in the brain. The antihypertensive therapy needs time to act, and it may take hours or even a day before the raised ICP shows signs of reduction after the start of antihypertensive therapy. It is, therefore, better to counteract an increase in ICP early by starting the ICP-reducing therapy as soon as possible after arrival at the hospital, regardless of the prevailing ICP, as recommended in the LC (3, 8).

The antihypertensive mechanisms originally used were beta-1 blockade and alpha-2 agonist. If not sufficiently effective, they were complemented with an angiotensin II antagonist (see below). The patient should normally be in a flat position with one pillow under the head. However, if there is a need to reduce CPP more than can be obtained from the antihypertensive treatment, the LC accepts moderate head elevation (15–20°). During calculation of CPP after head elevation, one must compensate for the increased vertical distance between the head and the heart.

Antihypertensive treatment was strongly questioned when introduced with the LC in 1992. It was entirely contrary to the general recommendations to keep CPP above 70 mmHg with vasopressors (4, 5, 46). This recommendation was changed in the Brain Trauma Foundation guidelines from 2007 (6) after publication of a study that showed that outcome was better with a relatively low CPP than with a high CPP, often obtained using vasopressors (49). It was later discussed whether the better outcome was an effect of the lower CPP or of less use of vasopressors, resulting in less ARDS (50).

Independently of this, US guidelines changed their recommendations in adults from a CPP of above 70 mmHg to a CPP of 50–70 mmHg (6), which is in the same range as has been recommended in the LC (3) and later also recommended in a study by Johnson et al. (51). A study by Elf et al. suggested that a CPP of 50–60 mmHg is acceptable if an optimal fluid therapy is used (52). Recommended CPP values in children are lower (3, 8). Note that CPP remains in the range of 60–70 mmHg with the LC in most adult patients in spite of the antihypertensive treatment, but somewhat lower CPP values than these can be accepted.

A CPP value in an unconscious patient after a TBI can be lower than what is normal in a healthy human being who is awake, especially if vasoconstrictors are not used. Just by lying in the supine position means that mean arterial pressure can be 15–20 mmHg lower than in upright position with the same perfusion, and even lower if the patient is sedated—as in the LC. In light of these considerations, it is reasonable that a CPP of 60–65 mmHg and even lower should be acceptable in s-TBI patients, if they are properly treated otherwise by avoiding hypovolemia, stress, and vasoconstrictors.

Note, however, that the CPP value alone does not reflect cerebral circulation, as it is also highly dependent on the blood volume status and the use of vasopressors and on the hyperadrenergic stress. Thus, a CPP of the lower range of 55–65 mmHg is only acceptable if normovolemia is maintained and no vasoconstrictors are used and with low adrenergic stress. Most CPP-targeted studies only present the CPP value without giving any information on blood volume and vasopressor status. This was the case also in those studies used in the latest Brain Trauma Foundation guidelines from 2016. In this version, the CPP recommendations have been changed from 50–70 mmHg to a lowest CPP of 60–70 mmHg (7).

As mentioned above, beta-1 blockade is an important antihypertensive component in the LC. It was initially strongly criticized by advocates of CPP-targeted guidelines. The criticism has more or less ceased after beta-blockade to head-injured patients received strong support from three independent human studies and one mouse study, which showed that beta-blockade was beneficial to the brain after s-TBI, with a significantly improved survival rate (53–56). Beta-blockade has also been shown to be protective on the cardiovascular system after TBI (57). We showed that beta-1 blockade has no direct influence on local cerebral hemodynamics after a TBI (58), and no side effects have been observed in TBI patients given beta-blockade. The LC is still the only TBI guideline to recommend beta-blockade.

It is well established that head-injured patients develop an adverse hyperadrenergic state with increase in proinflammatory sympathetic discharge and catecholamine release (42, 59). Alpha-2 agonists have become more and more popular as sedatives in general intensive care units and they damp a hyperadrenergic state as shown after cerebral ischemia (60). They are, however, still not recommended as a general drug for head-injured patients, except in the LC (3, 8, 9). Alpha-2 agonists effectively reduce blood pressure by their antisympathetic and sedative effects. They showed neuroprotective effects in an *in vitro* model of traumatic brain injury (61). An alpha-2 agonist decreases plasma catecholamine concentration and improves outcome from incomplete ischemia in the rat (62) and has no direct influence on local cerebral hemodynamics after TBI (58).

Most studies investigating the effects of alpha-2 agonists have analyzed the less selective catapressan. There are reasons to believe, however, that the newer, more selective alpha-2 agonist dexmedetomidine at a dose of 0.5–1.5 μg/kg/h is a better choice by minimizing vasoconsstrictor effects of a simultaneous alpha-1 stimulation. Note that much higher doses than those recommended in the LC both for catapressan and dexmedetomidine should be avoided as they may cause adverse vasoconstrictor effect. We still lack information about any side effects of alpha-2 agonists in the doses that are recommended in the LC.

Angiotensin II blockade is used as an antihypertensive drug in intensive care and can be an effective complement to beta-1 blockade and alpha-2 agonists in head-injured patients if arterial blood pressure is still too high. It reduces blood pressure with only a limited effect on the cerebral circulation (63, 64). It may also be beneficial by counteracting angiotensin II-induced inflammation (65) and also by reducing vascular permeability, as shown for glomerular permeability in the rat (66). However, there are no studies specifically analyzing the effect of this drug on patients with s-TBI.

No doubt, a markedly raised ICP (above 20–25 mmHg) is a severe negative sign. A raised ICP due to brain edema is more unfavorable than when ICP is increased due to temporary cerebral vasodilation.

## Principles for Perfusion of the Penumbra Zone

Brain cells are the most sensitive cells to hypoxia in the body. A s-TBI patient suffers from various degrees of compromised circulation in the brain. In most cases, there will be one or more severely hypoxic contusion areas, most of the cells of which will not survive independent of therapy. The area just outside the contusions, normally called the penumbra zone, is hypoxic but not dead—and has the potential to survive, especially in its outer (borderline) areas. The risk of severe hypoxia and cell death in less injured parts of the brain outside the penumbra zone is surely smaller. It is reasonable to believe that the degree of impairment of oxygenation of the penumbra zone is essential for outcome. Measures to improve oxygenation of the most injured parts of the penumbra zone would, therefore, be expected to improve outcome.

Oxygenation of the penumbra zone in the injured brain is dependent on the hemoglobin concentration and blood flow to this area. The flow of blood through a vessel is controlled by its vascular resistance and by the perfusion pressure. A high perfusion pressure accomplished by increase in arterial pressure with vasoconstrictor therapy has been the predominant measure in alternative guidelines, with the specific purpose of ensuring adequate perfusion of the injured brain (4, 5, 46). No doubt perfusion of parts of the brain outside the most injured parts of the penumbra zone is dependent on the perfusion pressure. As explained below, this may, however, not always be the case in the most injured parts of the penumbra zone.

**Figure 5:**
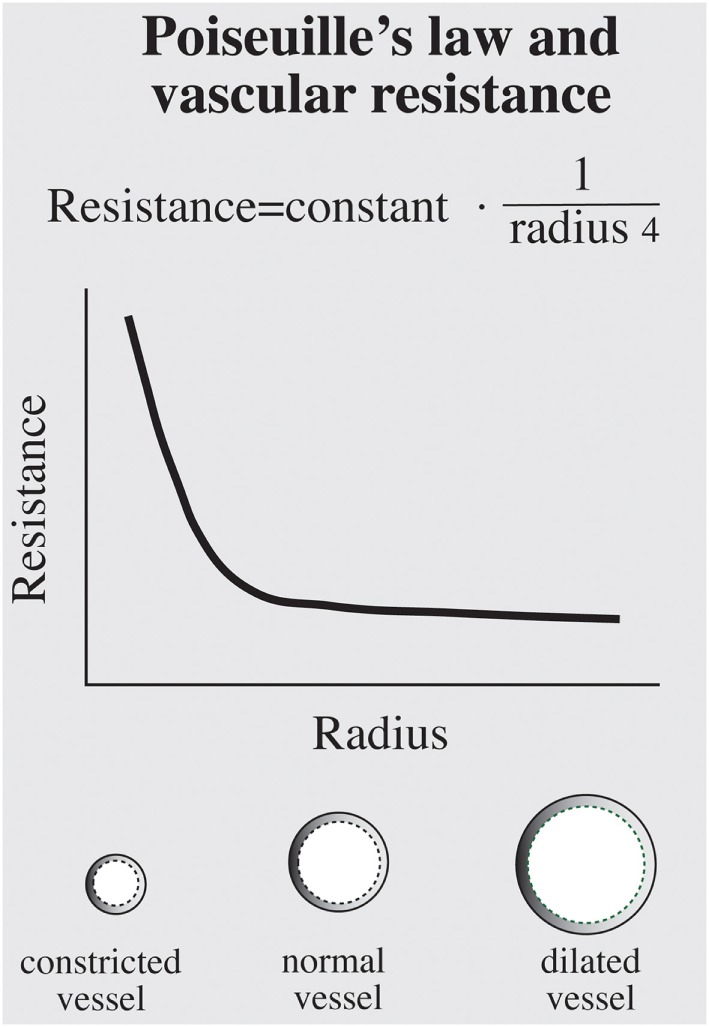
The relation between the flow resistance (*R*) and radius (*r*) of a vessel according to the law of Hagen–Poiseulle. As seen, this is a very unlinear relation (*R* = constant × 1/*r*^4^), which means that the effect of a vasoconstrictor is dependent on the initial vascular resistance. The vasoconstriction induced by a vasoconstrictor will be larger in the penumbra zone with a trauma-induced initial vasoconstriction than in less injured areas.

The relation between vascular resistance and vessel radius is formulated by the law of Hagen–Poiseuille (resistance = constant/radius^4^). This very unlinear fourth power relation means that the constrictor effect of a vasoconstrictor stimulus is dependent on the initial radius, as shown in Figure 5.

From a normal radius (in less injured parts of the brain), there will be just a moderate decrease in flow when influenced by vasoconstrictors, and the flow may even increase if the blood pressure is increased at the same time. In contrast, a vasoconstrictor stimulus in the most injured parts of the penumbra zone—with the radius of the vessels already reduced by the trauma—may result in a marked reduction in blood flow, even though there is a simultaneous increase in arterial blood pressure.

The penumbra zone most likely lacks myogenic response (and autoregulation), but it can still respond to alpha-1 stimulation from release of catecholamines through hypovolemia-induced baroreceptor reflex activation or infused catecholamines (67). As mentioned in the Section “Blood Volume” below, vasoconstrictor therapy also has adverse effects by reducing plasma volume, which, if not compensated for, will cause further activation of the baro receptor reflex and further release of catecholamines. There are indications that high catecholamine levels in TBI patients (endogenously or after noradrenalin infusion) lead to worse outcome in s-TBI patients (59).

Vasopressors such as noradrenalin, and also phenylephrin and vasopressin, are hypertensive drugs that are still used in alternative guidelines to increase blood pressure in TBI patients. For example, in the SAFE-TBI randomized study in Australia, high doses of noradrenaline were given to maintain CPP above 70 mmHg (68).

As discussed above, the use of vasopressors may reduce circulation, in the most injured parts of the penumbra zone (69), but to some extent also in less injured parts of the brain. Brassard et al. (70) concluded that there is a great risk that infusion of noradrenalin at 0.1 µg/kg/min or higher would negatively affect cerebral oxygenation. As suggested above, there is also a great risk that, through its CPP-increasing effect, noradrenalin increases brain edema. Finally, noradrenaline is a proinflammatory substance with permeability-increasing properties, and it may trigger the development of ARDS (49, 50, 71).

The need for vasopressors is significantly reduced when one accepts the moderate CPP values used in the LC, providing a simultaneous optimal fluid therapy as described under Section “Blood Volume” below. Vasopressors should be avoided, but may be necessary to maintain blood pressure at an adequate level in selected patients with heart failure, multiple injuries, or systemic inflammatory response syndrome (SIRS).

The risk of compromised circulation when treating the patient with antihypertensive drugs according to the LC is small, provided that there is an adequate blood volume substitution therapy and avoidance of vasopressors. This statement found support in a clinical microdialysis study with the microdialysis catheter placed in the penumbra zone. This study showed that TBI patients treated according to the LC could accept a CPP down to 50 mmHg without worsening of the hypoxia (72). Another clinical microdialysis study on patients with s-TBI and raised ICP, who were treated according to the LC, showed a gradual trend of normalization in the penumbra zone of the lactate/pyruvate ratio and glycerol concentration from raised levels. These microdialysis data indicate improved oxygenation and less cell derangement (73), which occurred in spite of the reduced CPP initiated by the antihypertensive treatment in the LC. The improved oxygenation and less cell derangement of the penumbra zone in these studies were most likely an effect of avoidance of hypovolemia, avoidance of vasoconstrictors and of reduced adrenergic stress.

In an attempt to improve the microcirculation of the penumbra zone pharmacologically, a low dose of prostacyclin has been an option in the LC since 1997, with a recommended dose of 0.7–1.2 ng/kg/min (34). Prostacyclin is an endogenous substance released from endothelial cells of the vacular wall. It is a potent inhibitor of platelet and leukocyte aggregation and inhibits their adhesion to the vascular wall and may thereby improve microcirculation. This hypothesis found support from a study in mice showing that prostacyclin reduces the contusion volume after brain trauma in a dose similar to that recommended in humans (74). Another study on the rat showed improved cortical perfusion with prostacyclin following brain trauma (75). Prostacyclin may increase the risk of bleeding, but only in higher doses than those recommended here. Prostacyclin may also reduce an increased vascular permeability (76).

**Figure 6:**
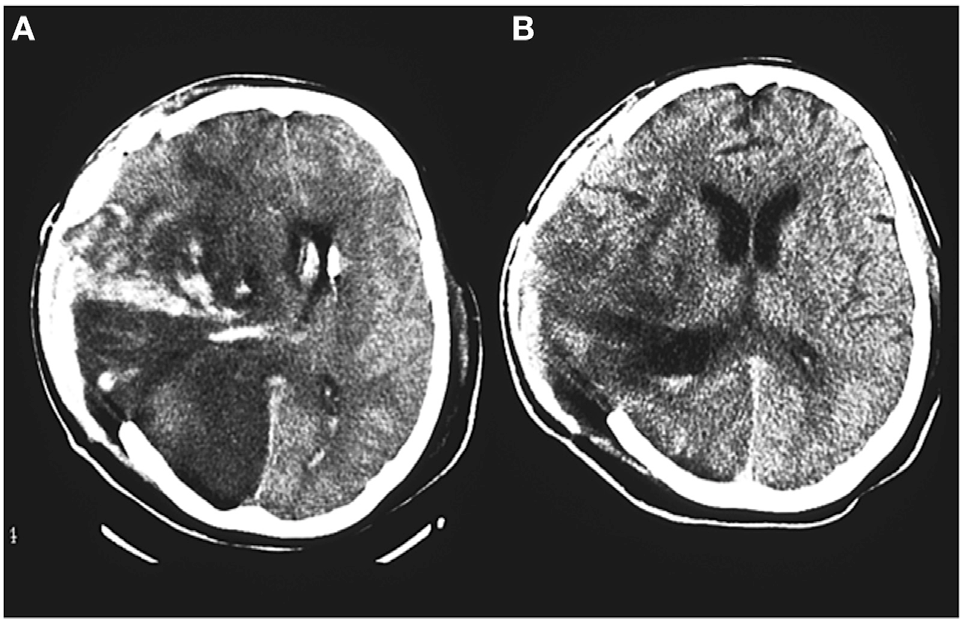
A CT scan of the brain from a 65-year-old woman treated with the Lund concept after a severe traumatic brain injury and craniectomy. **(A)** A CT scan before start of prostacyclin infusion. **(B)** A CT scan after 4 days of prostacyclin infusion (1 ng/kg/min i.v.). There are signs of improved perfusion of an area with compromised perfusion on the right side of the brain.

Two clinical microdialysis studies in s-TBI patients have shown improved oxygenation of the penumbra zone by prostacyclin (77, 78). That prostacyclin improves perfusion of hypoxic areas in a s-TBI patient has also found support from CT scans. Figure 6 illustrates such an example from an s-TBI patient showing a CT scan just before and another CT scan 4 days after the start of prostacyclin. Maybe it is not a coincidence that one of the best outcome results published from s-TBI patients included treatment with prostacyclin (10). Apparently, prostacyclin may be beneficial in s-TBI patients, but more clinical studies are needed for better evaluation of its composed effect in these patients.

These two prostacyclin studies (77, 78) and the two microdialysis studies presented above (72, 73) illustrate the value of the microdialysis technique in head injury research.

## Blood Volume

The traumatized brain suffers from an initial primary injury followed by a secondary injury. The secondary injury includes an inflammatory response with release of a host of cytokines and a profound acute-phase response, which is thought to contribute to disruption of the BBB and membrane damage of brain cells (19, 20, 79–82). Release of inflammatory substances from the injured brain may also cause SIRS with a general increase in systemic transcapillary leakage of fluid and proteins, and development of hypovolemia (83). This may explain why hemodynamic instability responsive to fluid resuscitation is common shortly after a traumatic brain injury, and also in the absence of systemic hemorrhage (84, 85).

An s-TBI, also without extracranial bleedings, results in a profound and fast decrease in plasma volume, as shown in a recent study on the cat. In this study, cats were exposed to a standardized fluid percussion brain trauma. The brain trauma resulted in 15% reduction in plasma volume, as measured 3 h after the trauma (86). In spite of the reduction in plasma volume, there was an increase in arterial blood pressure, most likely due to a trauma-induced hyperadrenergic state, showing that blood pressure is an unreliable parameter for evaluation of hypovolemia after a brain trauma.

In a study by Rise et al. (87) on anesthetized pigs, it was found that just a moderate hypovolemia, which had no adverse effects in normal pigs, resulted in compromised brain circulation after a brain injury. These authors suggested that alpha stimulation *via* activation of the baroreceptor reflex contributed to the compromised circulation (67, 87). A mechanism based on the law of Hagen–Poiseulle, as described under the section “Principles for Perfusion of the Penumbra Zone” above, may explain why the sensitivity to adrenergic vasoconstriction in the penumbra zone is increased after a brain trauma (Figure 5). This supports the hypothesis adopted in the LC that avoidance of hypovolemia is essential for good outcome after a brain injury.

## How to Prevent Hypovolemia

If the patient suffers an extracranial hemorrhage in combination with the head injury, the bleeding must be stopped and possible replacement of the blood loss by erythrocyte and plasma volume transfusion must be considered. If there is no extracranial hemorrhage, hypvolemia will still develop by transcapillary leakage. The degree of hypovolemia can be reduced by infusion of plasma volume expanders, and also by measures that counteract transcapillary leakage. Possible physiological mechanisms that counteract transcapillary leakage and physiological considerations regarding the effectiveness of various plasma volume expanders will be discussed below.

Even though there are difficulties in estimating the exact degree of hypovolemia, it can be roughly estimated with standard methods— such as by analyzing the arterial pulse pressure curve configuration, observing the blood pressure response upon a bolus infusion, or leg elevation. Note that the probability of hypovolemia is greater with low hematocrit than with normal hematocrit (see “Erythrocyte Transfusion” below). Most s-TBI patients will develop hypovolemia if not properly treated.

**Figure 7:**
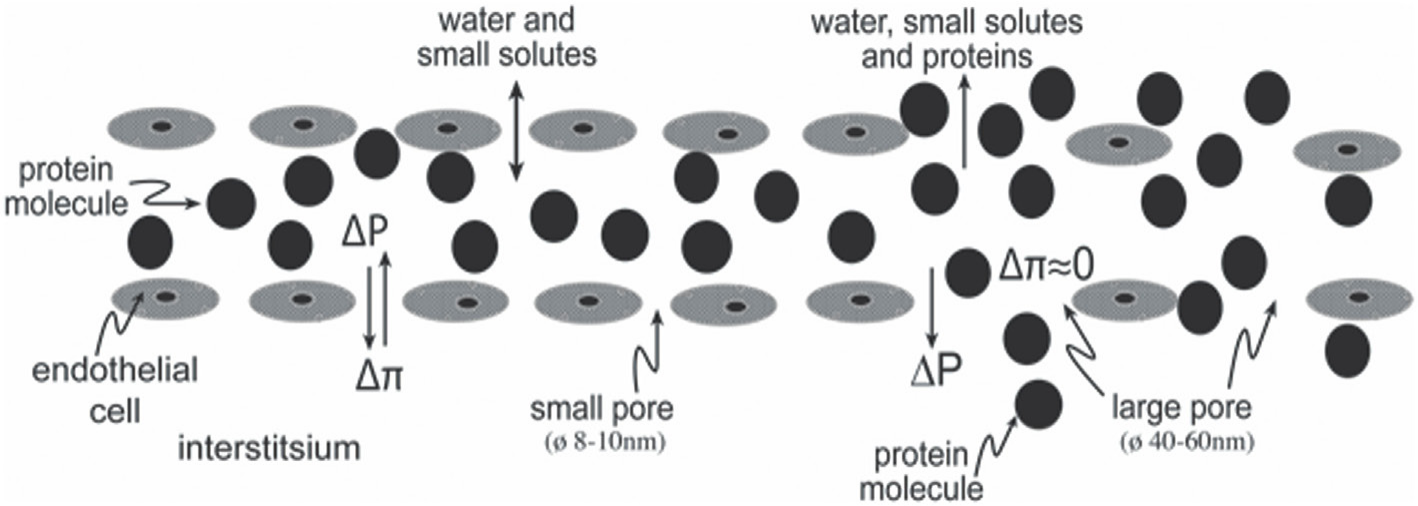
Transcapillary fluid exchange across a capillary outside the brain, according to the 2-pore theory. The illustration shows the large number of small pores across the whole capillary membrane that are permeable only to water and small molecules, such as electrolytes, and the much fewer large pores at the end of the capillary network and in venules, which are also freely permeable to proteins. No erythrocytes are shown. Due to the same oncotic pressure on both sides of the large pores, there is no oncotic absorbing force across the large pore (Δπ = 0). This means that the transcapillary hydrostatic pressure (ΔPc) is the only force across the large pores, creating a convective protein-rich volume flow through them. This passive transcapillary volume flow will increase after an increase in arterial pressure, increasing the risk of protein loss and hypovolemia. There will be an increase in the number of large pores after trauma, which will increase the transcapillary loss of proteins. This theory means that protein loss is a passive mechanism, mainly through convection. For details, see Ref. (88). Partly reproduced from Ref. (8) with permission.

Nowadays, the 2-pore model is a generally accepted model to explain transcapillary fluid exchange. It means that the capillary membranes outside the brain consist of a large number of small pores along the whole capillary membrane and only permeable for small solutes, and less frequent larger pores also permeable for larger molecules such as proteins, at the end of the capillary network and in venules (88). A schematic illustration of the 2-pore model is shown in Figure 7. All the pores are situated between the endothelial cells.

The two Starling forces, the hydrostatic and the oncotic transcapillary pressures, control fluid exchange through the small pores. The mechanisms that control fluid flow across the large pores are somewhat different. Due to the free permeability of proteins through the large pores, there will be about the same oncotic pressure on the intravascular and interstitial side of the pore. This means that the hydrostatic pressure is the predominant pressure force in the large pores, creating a non-energy-dependent passive filtration. Proteins will follow the fluid stream to the interstitium through the large pores, mainly by convection (88), and transcapillary loss of proteins occurs passively without energy-dependent active trancytosis.

**Figure 8:**
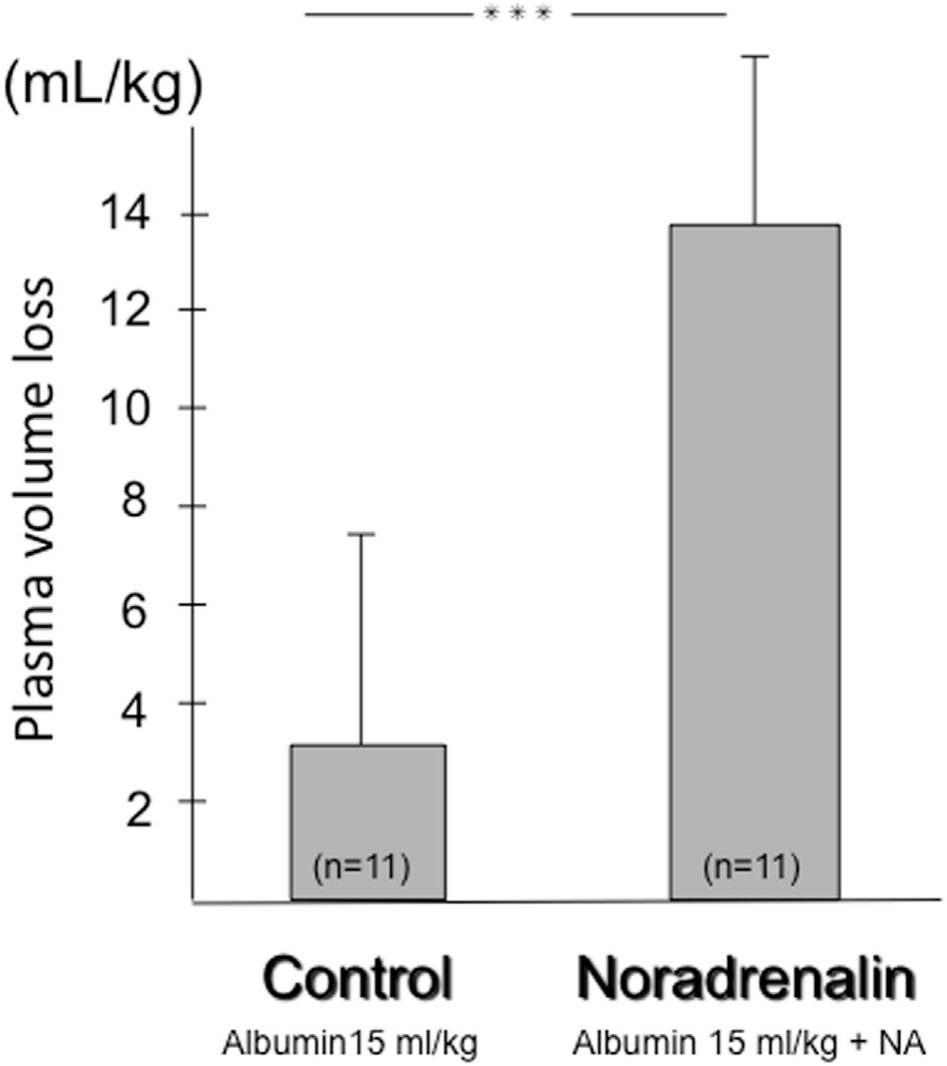
Effects on plasma volume in the septic rat of a bolus infusion of 5% albumin of 15 mL/kg, measured 2.5 h after the infusion in a control group with baseline mean arterial blood pressure and in a group where mean arterial blood pressure was increased by 12 mmHg using noradrenalin infusion. The loss of plasma volume was significantly greater in the noradrenalin group (14 mL/kg as opposed to 3 mL/kg), with loss of the main part of the infused volume. Data from Ref. (89), with permission.

The 2-pore model means that an increase in hydrostatic capillary pressure will increase transcapillary loss of fluid and electrolytes through both small and large pores, while transcapillary loss of proteins mainly occurs *via* convection through the large pores. This hypothesis was confirmed experimentally in a study on rats with SIRS, showing a significant loss in plasma volume following an increase in arterial blood pressure through noradrenaline infusion (Figure 8) (89). In this study, a plasma volume loss of about 3 mL/kg was measured 2.5 h after an albumin transfusion of 15 mL/kg in the control group with baseline arterial pressure, as compared to a corresponding loss of about 14 mL/kg when the mean arterial pressure was increased by about 12 mmHg by noradrenalin infusion. That there is an increase in plasma volume loss following noradrenalin infusion has also found support in a clinical study on patients with a large thoracic surgical trauma (90). Hydrostatic capillary pressure increases after noradrenalin infusion not only because of an increase in arterial pressure but also because of postcapillary venular vasoconstriction. The permeability-increasing effect of noradrenalin may also contribute to the plasma fluid loss.

## Fluid Substitution

Optimal fluid substitution is important for s-TBI patients, to prevent hypovolemia. As mentioned above, there is a risk that s-TBI patients develop hypovolemia very soon after the trauma, even if there is no extracranial bleeding (86). Isotonic crystalloids (e.g., saline or ringer lactate) and albumin are currently the only plasma volume expanders that can be recommended for s-TBI patients. At low hemoglobin concentration, blood erythrocyte transfusion will also increase the blood volume (see Erythrocyte Transfusion below).

Crystalloids are poor plasma volume expanders, as only 20–30% of the infused volume or even less will stay intravascularly. The rest (70–80%) will reach the interstitial space within 20 min after the infusion, contributing to general systemic tissue edema (91, 92). If only a crystalloid solution is used, there is a need for large fluid volumes to prevent hypovolemia.

There may also be a significant passive distribution of crystalloid fluids to the interstitium of the injured brain with a disrupted BBB. The use of crystalloids as plasma volume expander, therefore, may increase brain edema and ICP, especially if hypotonic solutions are used (93, 94). Still, infusion of moderate volumes of crystalloid solutions is important to maintain the fluid balance with normal urine production. Saline is the most common crystalloid used for s-TBI patients, but ringer lactate is an alternative. In larger volumes saline may lead to adverse hyperchloremic metabolic acidosis. A more balanced crystalloid solution may be a good choice.

Albumin, in combination with saline, has long been a standard plasma fluid expander in TBI patients. Apart from the absorbing effect of the increase in plasma oncotic pressure, which might reduce brain edema (95), albumin has a plasma-expanding effect, preventing hypovolemia, and thereby improving microcirculation in the penumbra zone. The plasma volume effect of albumin will, however, be reduced by the increase in transcapillary leakage of proteins, which may occur after s-TBI. The rate of this leakage, called the transcapillary escape rate, is normally 5–6% of the total plasma volume per hour, but can at least double after trauma and during systemic inflammation. The leaked albumin is transferred back to the circulation *via* the lymphatic system. When the leakage is greater than the lymphatic recirculating capacity—due to increased protein leakage or reduced lymphatic capacity—there will be a reduced protein concentration in plasma and interstitial accumulation of proteins. Simultaneously with their use as thrombosis prophylaxis, active physiotherapy and intermittent pneumatic leg compression may increase the recirculating capacity of the lymphatic system in arms and legs.

A retrospective outcome study on s-TBI patients showed that the concentration of albumin in plasma decreased considerably from normal values in the first days after trauma, and that low albumin levels were predictors of bad outcome (96). The authors suggested that low albumin levels are potential for albumin treatment. There have been several smaller single-center studies giving support for the use of albumin in s-TBI patients (97, 98). A study in rats showed that the use of albumin resulted in less brain edema after a head trauma than the use of saline in corresponding plasma-expanding doses (99). Albumin has been found to improve organ function in critically ill hypoalbuminemic patients (100). Another study in rats showed that albumin infusion improved systemic microcirculation and global hemodynamics and attenuated the inflammatory response to reperfusion by reducing rolling of leukocytes (101).

Only one study, the randomized SAFE-TBI study in Australia and New Zeeland, has found a worse outcome in s-TBI patients with albumin than with saline (68). The worse outcome with 4% abumin than with saline in that study was suggested to be an effect of raised ICP, without giving any more specific explanations (102). The SAFE-TBI study, however, has been called into question (103–106). It was a subgroup analysis involving 321 patients selected from an intensive care material of 7,000 patients. Subgroup analysis can be criticized because of the risk that the two groups may differ at baseline (107), as was also to the case in this study regarding baseline ICP and the number of patients with age above 55 years, both differences favoring the saline group. What is more important when criticizing this study is the use of hypotonic albumin solution of 255–260 mosm/L (whereas normal plasma osmolality is 290–300 mosm/L). It has been well established that hypotonic solutions are contraindicated in s-TBI patients, due to the risk of development of brain edema (93, 94). Ertmer and Van Aken (105) declared that the colloid compound was not the deleterious factor in the SAFE-TBI study, but that the study just confirmed that hypotonic solutions are deleterious in TBI patients. It was concluded by Ioannidis that evidence from trials, no matter how impressive, should be interpreted with caution when only one trial is available (108), a statement applicable for the SAFE-TBI study.

Considering the critic raised and the unexpected results of the SAFE-TBI study, it is a reasonable assumption that this study alone cannot be used to question the use of albumin in s-TBI-patients.

Severe traumatic brain injury patients require an effective plasma volume expander to avoid hypovolemia, but currently there is no such optimal plasma volume expander available. For the reasons described above, the use of a crystalloid as the only plasma volume expander to maintain normovolemia must be seen as a theoretical drawback for TBI patients for reasons described above. A crystalloid such as saline in combination with albumin should be a more reasonable choice. So far, there are no reasons to change the recommendations in the LC of a mixture of isotonic albumin (preferably 20% albumin) and saline as plasma volume expanders to s-TBI patients (3, 8, 9). It should be a reasonable goal to maintain albumin concentration of at least 32 g/L.

## Erythrocyte Transfusion

Erythrocytes contribute to a large proportion (normally 40%) of the intravascular volume. This means that there is a larger intravascular volume to be replaced by plasma volume expanders, to maintain normovolemia, at a low hemoglobin concentration than at a higher hemoglobin concentration. Erythrocytes may also reduce plasma volume leakage, as has been shown in studies on dogs and rats (109, 110). A hemoglobin concentration far below normal may even mean difficulties to maintain normovolemia.

Several studies have shown improved oxygenation of the brain after red blood cell transfusion (111–114). Considering the hypoxic penumbra zone, patients with s-TBI may represent a population of individuals who are particularly susceptible to anemia and hypovolemia. It is, therefore, reasonable to believe that s-TBI patients cannot be compared with general intensive care patients regarding possible beneficial effects of blood transfusion. There are theoretical arguments for giving blood transfusions specifically to s-TBI patients at a low hemoglobin concentration, with the purpose of improving oxygenation of the penumbra zone, to reduce cytotoxic brain edema and to maintain normovolemia. That’s why erythrocyte transfusions of up to a hemoglobin concentration of above 110 g/L has been recommended previously to s-TBI patients in the LC, and always with leukocyte-depleted blood (3, 8). As described below some adjustment of recommended hemoglobin level toward somewhat lower levels has been done.

A study by McIntyre et al. showed a not fully significantly improved outcome in s-TBI patients with a liberal transfusion strategy (a 60-day mortality of 17 vs 13%) (115), and these results even though the blood was not leukocyte-reduced in this study. The literature shows varying results regarding the effects on outcome after blood transfusion in s-TBI patients, as summarized in a recent review (116). Despite the potentially improved oxygenation of the penumbra zone and less risk of hypovolemia, some studies have indicated worse outcome after blood transfusion, whereas others have not (117, 118). The quality of the blood may be a factor of great importance. Perhaps the most negative characteristic of transfused red blood cells is the leukocyte content. For example, leukocytes have been recognized to be a strong contributor to a number of adverse effects of blood transfusion. Leukocytes are proinflammatory, and outcome in the intensive care unit is improved and fever reduced when using leukocyte-depleted blood instead of non-leukocyte-depleted blood (119–122). Also, the storage time is of importance for the quality of blood (123). In most of the erythrocyte transfusion studies presented so far on s-TBI patients, non-leukocyte-depleted blood has been used. Interpretation of the results may also be difficult when the low hemoglobin concentration and the use of blood transfusion reflect the degree of illness (117), and the amount of blood transfused reflects severity of injury. For example, in the study by Salim et al. (117), the patients given blood were older, were more severely injured from the start, and had a lower rating on the Glascow Coma Scale.

The current knowledge regarding the effect on outcome of erythrocyte transfusion in patients with TBI is highly conflicting. We still lack any strong indications that erythrocyte transfusion is beneficial for outcome in s-TBI patients, in spite of the fact that many studies have shown improved oxygenation and that anemia with hemoglobin below 9 g/L is a predictor of bad outcome (26), and that blood is a good blood volume expander. The improved oxygenation and our non-scientific experience of a hemodynamically more stable patient with a tendency of reduced ICP after blood transfusion cannot be enough for general acceptance of erythrocyte transfusion. We can never overcome the fact that blood transfusions are transplantations from other individuals, with many still unknown adverse components. That leukocyte-depleted blood was not used in most studies may have been of importance for the outcome in these studies. There has been no randomized study of the optimal hemoglobin concentration in neurocritical care. Due to the lack of convincing scientific support for our original hypothesis in the LC of improved outcome with a relatively liberal use of transfusion with leukocyte-depleted blood, we can recommend a somewhat more restrictive use of blood to s-TBI patients. A new target hemoglobin level could be 105–110 g/L, always with leukocyte-depleted blood.

## Plasma-Sparing Components of the LC

Some of the components of the LC (denoted I–VI below) may help to reduce the transcapillary leakage of plasma fluid and plasma proteins, and thereby the need for plasma volume expanders and counteracting hypovolemia.

The 2-pore model (Figure 7) means that transcapillary loss of plasma fluid and plasma proteins is highly dependent on the hydrostatic capillary pressure, as also confirmed both experimentally (89) (Figure 8) and clinically (90). If so, the use of antihypertensive therapy (I) and avoidance of vasopressors (II) will reduce the need for plasma volume expanders. As discussed above, the use of albumin (e.g., 20% isotonic solutions) (III) and avoidance of hemoglobin concentrations that are too low (IV) may help to counteract transcapillary loss of fluid.

A study in septic guinea pigs showed that a slow rate of infusion of albumin has better plasma-expanding effect than a fast infusion rate (124). If so, a low rate of infusion of albumin may improve its plasma-expanding effect (V).

Frequent physiotherapy and intermittent pneumatic leg compression with antithrombotic stocks may not only counteract deep leg thrombosis but may also help to bring back interstitial fluid to the circulation by increasing the capacity of the lymphatic recirculating system in the legs (VI). Components I–VI have been presented in Table 2.

**Table 2:** Potential measures adopted in the Lund concept to reduce transcapillary leak and the need of albumin (and crystalloids) as plasma volume expander.

(I) Avoidance of high arterial pressure with antihypertensive drugs
(II) Avoidance of vasopressors
(III) The use of albumin as plasma volume expanders, preferably 20% solutions
(IV) Avoidance of low hemoglobin concentrations
(V) Low infusion rates of albumin
(VI) Physiotherapy and antithrombotic pneumatic leg compressions

The effectiveness to spare plasma for each of these six components is not evaluated, but our experience supports that these measures taken together reduce the need for albumin.

## Ventilation

All patients with an s-TBI treated with the LC are mechanically ventilated. Volume-controlled ventilation is preferred to minimize variation in arterial ${\text{P}}_{{\text{CO}}_{\text{2}}}$ during secrete stagnation. In line with the general goal in the LC of maintaining normality as far as possible, the TBI patients are normoventilated, keeping arterial ${\text{P}}_{{\text{CO}}_{\text{2}}}$ within a normal range (4.5–5.0 kPa). Underventilation should be avoided because of the risk of cerebral hyperemia with increase in intracranial blood volume and ICP. Hyperventilation, on the other hand, means a risk of vasoconstriction and increased hypoxia, especially in the penumbra zone and is not recommended (125, 126). Hyperventilation down to arterial ${\text{P}}_{{\text{CO}}_{\text{2}}}$ of 25 mmHg for the purpose of reducing ICP is still accepted in some alternative guidelines (e.g., the Brain Trauma Foundation guideline from 2016) (7).

Oxygen concentration in the ventilator should be set to give a normal arterial ${\text{P}}_{{\text{O}}_{2}}$ of around 12 kPa, helping to optimize oxygenation of the penumbra zone. Higher arterial ${\text{P}}_{{\text{O}}_{2}}$ should be avoided because of risk of hyperoxic cerebral vasoconstriction and hyperoxic lung damage (127, 128).

High-dose barbiturate and noradrenalin treatment may trigger pulmonary insufficiency and fever, as mentioned under Section “Sedation” below and under section “Principles for Perfusion of the Penumbra Zone” above (50) and was not used in the LC. PEEP is obligatory to prevent atelectasis and is safe for the brain, as discussed above under Section “Hemodynamic Consequences of the Rigid Cranium.” Tracheostomy can be considered in selected cases.

Intermittent cautious moderate bagging under ICP control and inhalation to prevent secrete stagnation and atelectasis have been recommended in the LC. Note that inhalation with beta-2 stimulating drugs may induce transient vasodilation with increase in ICP and decrease in blood pressure—and, if so, the dose should be reduced to half the dose or less when starting inhalation next time.

## Active Cooling

The concept of active cooling of s-TBI patients was discussed in detail in a recent review (129). Active cooling of patients with TBI was first described by Fay in 1945 (130) and has become a major area of research during the last 2–3 decades. Based on the convincing neuroprotective effect of hypothermia under hypoxia as illustrated by case reports showing good recovery after drowning in cold water (129), great expectations have been raised about active cooling as a breakthrough in s-TBI patients (131). In spite of this, general active cooling has never become a component of the LC, mainly because of the pathophysiological arguments presented below.

Even though cooling is neuroprotective and may improve outcome after a general brain hypoxia, the situation is different after s-TBI. The traumatized brain often suffers from compromised circulation and hypoxia in and around its most injured areas, the penumbra zone. It may, therefore, be extra sensitive to the hyperadrenergic stress induced not only by the brain trauma itself (42–44) but also by that induced by active hypothermia. Active cooling means that the temperature of the body becomes lower than the value set in the biological thermostat. This difference creates a marked adrenergic stress superimposed on the head trauma-induced stress, with shivering, increased sympathetic discharge, and catecholamine release. The hypothermia-induced stress may further compromise circulation of the penumbra zone. The powerful hypothermia-induced adrenergic stress is aimed at resetting the body temperature to the value set in the biological thermostat of the brain. This view has found support from studies showing a significant reduction in brain oxygenation following active cooling simultaneously with an increased metabolism of the most injured parts of the brain (132–134).

There are other complications attributed to hypothermia, such as coagulopathy, cardiovascular complications, and especially, pneumonia (129, 135). A Cochrane analysis pointed out the risk of pulmonary complications with active cooling (136).

These pathophysiological principles and consequences may explain why none of the randomized high-quality hypothermia studies performed during the last —two to three decades in s-TBI patients have shown any beneficial effects on outcome; some have even indicated that active cooling is detrimental for outcome. The randomized hypothermia studies in s-TBI patients performed are presented below.

The well-designed pediatric studies by Hutchison et al. (137) and by Adelson et al. (138) showed no significant differences in outcome between the hypothermia groups and the corresponding control groups. In the Hutchinson study, there was a significantly higher mortality in the hypothermia group in a subgroup of patients over 7 years of age—with a mortality rate of 21% as compared to 12% in the control group.

The two studies by Clifton et al. (139, 140) can also be classified as high-quality studies. There was no significant difference in mortality between the hypothermia group and the normothermia group in these studies, but there was a tendency of worse outcome in the hypothermia group with a longer hospital stay and more complications.

That hypothermia lowers a raised ICP is well known from most hypothermia studies [e.g., Ref. (131)]. This is most likely an effect of hypothermia-induced vasoconstriction, with a simultaneous reduction in cerebral blood flow and blood volume. This experience initiated a large multicentre European study evaluating the effect on outcome of hypothermia to 33–35°C in patients with an ICP above 20 mmHg (134). In this well-performed study, as expected, they found that ICP decreased with hypothermia, but there was no improvement in outcome. If anything there was a tendency of worse outcome in the hypothermia group, and the study was interrupted prematurely. Later analysis of the material from this study also showed that hypothermia reduced oxygenation of the brain.

To summarize, the best hypothermia studies taken together from the last two decades have shown that hypothermia does not improve outcome or, if anything, they have shown a tendency of worse outcome in s-TBI patients. This conclusion finds support from a Cochrane study, which concluded that head trauma patients treated with hypothermia, were slightly more prone to die (136). Active hypothermia is no longer recommended in the Brain Trauma Foundation guidelines from 2016. Therefore, the recommendation made in the LC 25 years ago that TBI patients should not be treated with active hypothermia (3, 8) has found strong support during the last two decades.

## Fever

It is believed that high fever is deleterious in TBI patients (141). It appeared that high fever in s-TBI patients was much less common in our intensive care unit after introduction of the LC, which may have several explanations. Pulmonary complications in terms of atelectasis and pneumonia were reduced by PEEP, by avoidance of vasopressors and especially by avoidance of high-dose treatment with barbiturate. Change from high calories parenterally to lower calories, mainly with enteral nutrition, may also counteract the development of fever (see “Nutrition” below). Paracetamol reduces fever by affecting the biological thermostat. The effect, however, is relatively small (temperature reduction by 0.5°), but is used in the LC.

Steroids might be an alternative to reduce fever by affecting the biological thermostat. Use of steroids to TBI patients, however, can be questioned after the CRASH study, which showed worse outcome in TBI patients (most of them without fever) treated with high doses of metylprednisolone (in total 22 g for 2 days) (142). As mentioned above, active general cooling is not a therapeutic component of s-TBI patients in the LC, but if temperature is persistently high (>39.5–40°C), it must be reduced in some way—and in this situation, a more long-term active cooling or a bolus dose of a steroid (e.g., metylprednisolon 0.25–0.5 g) may be life-saving.

## Osmotherapy

Osmotherapy with mannitol has been used since the beginning of the 1960s as the main treatment for a raised ICP and is still a main component of most s-TBI guidelines. Hypertonic saline has become an alternative osmotic drug during the last 20 years. In spite of its use for more than 55 years, and more than 155 publications, the scientific world has failed to come up with reliable beneficial data on outcome related to osmotherapy.

A Cochrane analysis did not find any beneficial effects on outcome of osmotherapy (143), a conclusion that was in agreement with that in a recent review on osmotherapy (144). The only studies showing favorable effects of mannitol on outcome appeared to be faked (145). Osmotherapy effectively reduces ICP, but mannitol especially has severe side effects in terms of renal and pulmonary failure, electrolyte disturbances, and a rebound increase in ICP after the mannitol infusion is terminated (143).

The rebound increase in ICP after the mannitol infusion is discontinued may be an effect of intracellular accumulation of mannitol in the brain creating an osmotic fluid filtration force when plasma mannitol concentration is reduced after the infusion is stopped. A rebound effect of mannitol has also been demonstrated experimentally on cat skeletal muscle enclosed in a plethysmograph (146). It can be speculated that the rebound effect could to some extent also be an effect of increased transcapillary pressure following the mannitol-induced reduction in ICP (see Decompressive Craniectomy below).

In combination with its side effects, mannitol treatment has the weakness of just treating the symptom (raised ICP) without attacking the pathophysiological mechanisms behind the symptom. There are apparently arguments against the use of osmotherapy, and especially mannitol. Except from acute prevention of brain stem compression, osmotherapy has not been a component of the LC. During the 25 years that LC has existed, no new information has been presented to support any change in the recommendation in the LC regarding osmotherapy (3). The need of osmotherapy in LC is reduced by other ICP-reducing measures, such as antihypertensive and sedative therapies.

## Decompressive Craniectomy

After some sceptisism during the 90th decompressive craniectomy has nowadays become included in most TBI guidelines. Early on, it became a component of the LC in patients with uncontrolled high ICP after other measures to reduce ICP had failed. Craniectomy reduces ICP by giving extra space to the swollen brain, and it may quickly prevent brain stem compression and death. Side effects with craniectomy may be reduced by considering its hemodynamic consequences, as clarified below with the help of Figure 2.

A reduction in ICP after decompressive craniectomy means an increase in transcapillary hydrostatic pressure. This will increase the transcapillary filtration, creating more brain edema and expansion of the swollen brain, especially in the cranial opening—as routinely seen on the CT scan. If not counteracted, the development of brain edema is a potential drawback of craniectomy with the risk of herniation, axonal stretch, and strangulation in the cranial opening (147, 148). As discussed below, brain edema after craniectomy can be reduced when considering hemodynamic aspects for development of vasogenic brain edema.

After craniectomy, there is often a sudden marked reduction in the raised arterial pressure simultaneously with a drastic reduction in ICP. This is often followed by a moderate increase in ICP (149). It can be speculated that this increase in ICP after cranietomy is an effect of the unbalance in the Starling fluid equilibrium following the initial lowering in ICP. The spontaneous reduction in arterial pressure in combination with an active treatment with antihypertensive drugs and treatment with albumin should counteract the development of brain edema in the cranial opening.

According to these theories, CPP should be kept at a relatively low level with antihypertensive treatment after craniectomy. This may explain the beneficial outcome after craniectomy in a study in which the LC therapy was used (149). In that study, there was no difference in outcome between non-craniectomized patients and craniectomized patients, even though the craniectomized patients had higher ICP initially.

These hemodynamic principles may give a hint as to why outcome results after craniectomy differ between studies. In a randomized study in Australia by Cooper et al. (150), unfavorable outcome was found after craniectomy, while a randomized study in Cambridge showed improvement in outcome after cranietomy, but at the expense of more disability (151). The study by Cooper et al. has been criticized for the low entry criterion for ICP of 20 mmHg and that the study gave no information about CPP and the use of vasopressors (150). It is well known that vasopressors are frequently used in TBI patients in Australia (e.g., the SAFE-TBI study) keeping CPP above 70 mmHg (68).

It seems that decompressive craniectomy may be a life-saving measure for prevention of brain stem herniation and death at an uncontrolled high ICP, if otherwise optimal treatment is used. Decompressive craniectomy is a component of the LC.

## Other Surgical Measures

Evacuation of hematomas and focal lesions and CSF drainage, also mean loss of transcapillary counter-pressure with the risk of a subsequent brain edema development, providing a disrupted BBB.

Removal of space occupying hematomas and superficial contusions will reduce a raised ICP and may improve outcome, and is accepted in the LC. The improved outcome most likely is not only an effect of a decrease in intracranial volume but also an effect of reduced release of toxic substances from hematomas and contusions (3).

**Figure 9:**
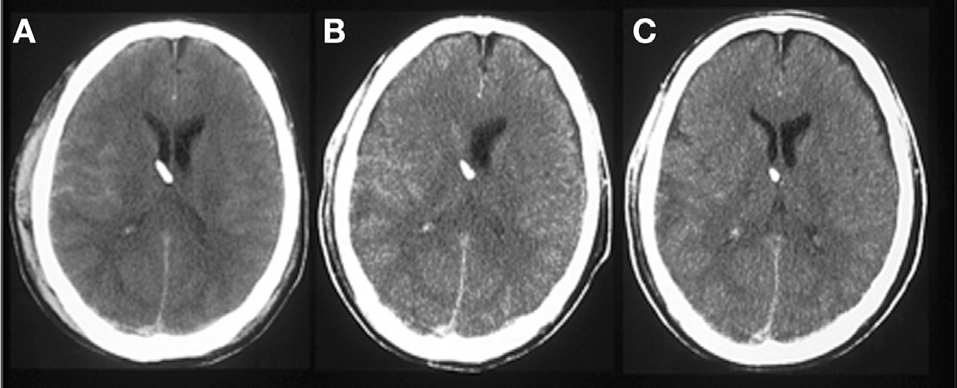
A CT scan of the brain from a 45-year-old man after a severe traumatic brain injury. **(A)** A CT scan before the start of drainage at an intracranial pressure of 32 mmHg. **(B)** A CT scan after 1 day of ventricular drainage, at a drainage level of 22 mmHg. **(C)** CT scan 2 days after drainage was stopped. Ventricular size was reduced by the drainage, and partly restored after the drainage was stopped. The figure supports the presented principles for transvascular fluid exchange in the injured brain with disrupted blood–brain barrier.

Ventricular drainage of CSF can sometimes be an effective way of controlling ICP. There is always a risk, however, that the volume of drained CSF is replaced by edema when transcapillary pressure is increased after reduction of ICP by the drainage procedure—with increased risk of ventricular collapse. Such a scenario is shown in Figure 9. CSF drainage was started at an ICP of 32 mmHg at a drainage level of about 22 mmHg (Figure 9A). After 1 day of drainage, there was a smaller ventricular volume (Figure 9B). The drainage was now closed and the ventricular volume was partly recovered about 2 days thereafter (Figure 9C). Figure 9 gives support for principles for transcapillary exchange in the injured brain as described under Section “Principles for Brain Volume Regulation” above.

Reduced ventricular volume during drainage can be prevented if intermittent drainage is used or if the drainage level is set at a level that is not too far below the initial ICP level (e.g., 2–4 mmHg), followed by a successive slow reduction over days. Ventricular sizes should be followed regularly with CT scan to discover incipient ventricular collapse. Ventricular drainage is also recommended in the last Brain Trauma Foundation guidelines (7), but the drainage level is not specified. The ventricular collapse effect following CSF drainage may explain why we still lack studies showing a beneficial outcome effect of ventricular drainage.

## Nutrition

At the time of introduction of the LC 25 years ago, there was a general recommendation that adult patients in intensive care should be given high-energy nutrition of up to 3,000 kcal every 24 h to counteract catabolism. To achieve this energy supply, large volumes of parenteral nutrition with lipids in combination with amino acids and glucose were used. Our metabolic measurements in adult s-TBI patients, however, showed a basal metabolism in these sedated, adrenocortically depressed, and artificially ventilated patients of only 1,200–1,300 kcal/24 h or less.

At that time, new ideas were being introduced that overnutrition should be avoided, as more energy than the basal need cannot be utilized in highly catabolic patients, creating fever. When we changed from high-energy, mainly parenteral nutrition to a more low energy, mainly enteral nutrition, we noticed less fever in our s-TBI patients. It is well known that parenteral overnutrition with fat can induce hemophagocytosis and fever (152) and that infections are more common in patients who are treated with parenteral nutrition than with enteral nutrition. Since then, our recommendation in the LC has been an initial energy supply from day 2 of 15–20 kcal/kg/24 h to the adult—mainly enteral nutrition, if necessary complemented with infusion of intravenous 5% glucose—later followed by a slow increase in the supply. This regime should be followed to prevent malnutrition and overnutrition. Children need more energy per kilogram. This relatively low-energy supply means that most of the nutrition can be given through the natural and more beneficial enteral form. If necessary to reach enough caloric supply, it can be complemented with glucose solutions with electrolytes. Avoidance of both overnutrition and malnutrition has become a generally accepted goal in the intensive care unit (153). Blood glucose should not be in the lower range and be kept between 6.0 and 8.5 mmol/L, if necessary with insulin, which is in agreement with the NICE SUGAR study (154).

## Sedation

Reducing general stress and the adrenocortical influence is an important component of the LC. Even unconscious TBI patients can be severely stressed, with increased blood pressure and ICP. Extra stress may occur during tracheal suctioning and from alarms and other noises in the room, wake-up tests, and insufficient sedation. The increased adrenocortical stress in TBI patients is reduced in the LC by the use of antihypertensive drugs, such as beta-blocker, alpha-2 agonist, and sometimes angiotensin II agonist and by avoidance of the use of catecholamines (see above). The stress is also reduced in the LC by sedation with, midazolam, and fentanyl—which also makes wake-up tests inexpedient in the LC (3).

Heavy sedation may have pulmonary side effects. Therefore, the degree of sedation should not be deeper than what is necessary to effectively reduce stress, and the seemingly unstressed patient should not be given aggressive sedation. The degree of sedation should be successively reduced in relation to the observed decrease in ICP with a change toward short-acting sedatives, such as propofol before start of the weaning phase. The depth of sedation aims at avoiding normal clinical signs of stress such as inoppropriate movements and coughing combined with an increase in ICP. Due to the anti-stress therapy, epileptic seizures are very rare or absent with the LC, as shown in a separate study (155). Prophylactic anti-convulsive treatment is, therefore, not a component of the LC. We normally do not measure depth of sedation with BIS or cEEG.

High-dose barbiturate treatment was common in TBI patients in our intensive care unit before we started with the LC in 1991–1992. At that time, we had severe complications in TBI patients in terms of pulmonary complications and frequent ARDS, and quite often very high fever. An experimental study in the cat showed that a high dose of barbiturates, except that it caused complete inhibition of autoregulation of blood flow, it caused an almost complete inhibition of the actin-myosin activity in vascular smooth muscle cells (156). A similar inhibition of spontaneous actin-myosin bronchial ciliae activity may result in a drastic inhibition of the self-cleaning capacity of the lung, with increased risk of ARDS and pneumonia. We cannot tell for sure whether such a mechanism contributed to the high frequency of ARDS, pneumonia, and fever in TBI patients at that time, but these lung problems were drastically reduced after high-dose barbiturate therapy was stopped. Barbiturates are still given in the LC in selected cases, but only as an extra sedation and to restrict a life-threatening high ICP—and only low doses are used (<2–3 mg/kg/h) for 2 days at most (3). No studies have shown improved outcome with barbiturate therapy (157).

Still, high-dose barbiturate treatment in doses below those giving burst suppression pattern is accepted in some alternative guidelines (7). Both barbiturate treatment and mannitol treatment suffer from the weakness that they just treat the symptom of raised ICP, without addressing the pathophysiological mechanisms behind the raised ICP.

## Summary

In the present review, I have described some hemodynamic principles, that may be of value in understanding various scenarios in the traumatized brain, and when formulating treatments for s-TBI patients. I have also discussed certain aspects of components such as temperature, ventilation, nutrition, osmotherapy, decompressive craniectomy, and sedation. Most of the principles discussed find support from physiological and pathophysiological principles and from clinical and experimental studies from our and other groups. Clinical confirmation of some components, such as liberal use of blood transfusion with leukocyte-depleted blood, is still lacking. For doses of the different drugs used (3).

## Author Contributions

The author confirms being the sole contributor of this work and approved it for publication.

## Conflict of Interest Statement

The research was conducted without commercial, financial, or other conflict of interests.
